# Assessing health-related quality of life in COPD: comparing generic and disease-specific instruments with focus on comorbidities

**DOI:** 10.1186/s12890-016-0238-9

**Published:** 2016-05-10

**Authors:** Margarethe E. Wacker, Rudolf A. Jörres, Annika Karch, Sarah Wilke, Joachim Heinrich, Stefan Karrasch, Armin Koch, Holger Schulz, Henrik Watz, Reiner Leidl, Claus Vogelmeier, Rolf Holle, for the COSYCONET-Consortium

**Affiliations:** Institute of Health Economics and Health Care Management, Helmholtz Zentrum München GmbH - German Research Center for Environmental Health, Comprehensive Pneumology Center Munich (CPC-M), Member of the German Center for Lung Research (DZL), Ingolstädter Landstr. 1, 85764 Neuherberg, Germany; Institute and Outpatient Clinic for Occupational, Social and Environmental Medicine, Ludwig-Maximilians-Universität München, Ziemssenstr. 1, 80336 Munich, Germany; Institute for Biostatistics, Hannover Medical School, Carl-Neuberg-Str. 1, 30625 Hannover, Germany; Department of Research & Education, CIRO, Hornerheide 1, 6085 NM Horn, The Netherlands; Institute of Epidemiology I, Helmholtz Zentrum München GmbH - German Research Center for Environmental Health, Comprehensive Pneumology Center Munich (CPC-M), Member of the German Center for Lung Research (DZL), Ingolstädter Landstr. 1, 85764 Neuherberg, Germany; Institute of General Practice, University Hospital Klinikum rechts der Isar, Technische Universität München, Orleansstr. 47, 81667 Munich, Germany; Pulmonary Research Institute at LungenClinic Grosshansdorf, Airway Research Center North (ARCN), Member of the German Center for Lung Research (DZL), Wöhrendamm 80, 22927 Grosshansdorf, Germany; Institute of Health Economics and Health Care Management, Munich Center of Health Sciences, Ludwig-Maximilians-Universität München, Ludwigstr. 28/RG, 80539 Munich, Germany; Department of Respiratory Medicine, University of Marburg, University Giessen and Marburg Lung Center (UGMLC), Member of the German Center for Lung Research (DZL), Baldingerstraße, 35043 Marburg, Germany

**Keywords:** Chronic obstructive pulmonary disease, Health-related quality of life, Utility, Comorbidity, Cohort study, Questionnaires

## Abstract

**Background:**

Chronic Obstructive Pulmonary Disease (COPD) influences different aspects of patient’s health-related quality of life (HRQL). While disease-specific HRQL instruments focus on symptoms and functional impairments, generic instruments cover a broader view on health. This study compares the generic EQ-5D-3 L and two disease-specific questionnaires (St.-George’s Respiratory Questionnaire (SGRQ-C), COPD Assessment Test (CAT)) in a comprehensive spectrum of COPD disease grades with particular attention on comorbidities and assesses the discriminative abilities of these instruments.

**Methods:**

Using data from the baseline visit of the German COPD cohort COSYCONET, mean HRQL scores in different COPD grades were compared by linear regression models adjusting for age, sex, education, smoking status, BMI, and low vs. high number of comorbidities or a list of several self-reported comorbid conditions. Discriminative abilities of HRQL instruments to differentiate between COPD grades were assessed by standardized mean differences.

**Results:**

In 2,291 subjects in COPD GOLD grades 1–4 EQ-5D-3 L utility, EQ-5D VAS, SGRQ, and CAT were found able to discriminate between COPD grades, with some limitations for the EQ-5D utility in mild disease. Both generic and disease-specific HRQL instruments reflected the burden of comorbid conditions. The SGRQ showed the best discrimination between COPD grades and was less influenced by comorbidities, while EQ-5D utility put a higher weight on comorbid conditions. For all instruments, psychiatric disorders and peripheral artery disease showed the strongest negative associations with HRQL.

**Conclusion:**

All HRQL instruments considered reflect considerable impairment of HRQL in COPD patients, worsening with increasing COPD grade and number of comorbidities. Findings may support clinical assessment, choice of HRQL instrument in future studies, and parameterization of decision-analytic models.

**Electronic supplementary material:**

The online version of this article (doi:10.1186/s12890-016-0238-9) contains supplementary material, which is available to authorized users.

## Background

Chronic Obstructive Pulmonary Disease (COPD) is a common progressive disease that is characterized by persistent airflow limitation and related to an enhanced chronic inflammatory response in the airways and the lung to noxious particles or gases [1]. Although preventable and treatable, COPD represents an important public health challenge which is projected to increase in coming years because of continued exposure to risk factors such as tobacco smoking, indoor and outdoor air pollution and the aging population. The World Health Organization predicted that COPD will become the fourth leading cause of death worldwide by 2030 [2].

Patients with COPD usually experience a decrease in their health-related quality of life (HRQL): COPD symptoms such as cough, dyspnea, and sputum production, as well as acute exacerbations of the disease, and comorbidities which are ubiquitous in COPD patients contribute to the overall severity of the disease [3–6].

HRQL as an important patient-reported outcome measure in COPD has gained attention in the last years both as an individual descriptive measure as well as an endpoint in clinical studies [7]. A large variety of generic and disease-specific HRQL instruments with proven validity and reliability is available to assess HRQL of COPD patients [8]. While disease-specific instruments focus on symptoms and functional impairments associated with COPD, generic instruments are more widely applicable irrespective of the underlying disease [7]. Therefore, they can be used to compare HRQL of diseased and healthy subjects or to compare the burden of different diseases. Some generic questionnaires are used as utility instruments e.g. for the calculation of quality-adjusted life years (QALYs) in health economic evaluations. Nevertheless, they may be less sensitive to special problems of a certain disease than disease-specific instruments.

This paper describes generic as well as disease-specific HRQL in a large cohort of patients with COPD comprising all disease grades and compares assessment properties and correlations of different HRQL instruments. HRQL instruments are analyzed regarding their ability to differentiate between COPD grades. Special attention is given to the impact of comorbid conditions in COPD patients on generic and disease-specific HRQL assuming that generic instruments are more suitable to reflect the impact of comorbidities on health status than disease-specific instruments. Furthermore, we analyze whether COPD and comorbidities have additive effects on HRQL measures and whether this is different for generic and disease-specific instruments.

## Methods

### Patients

This cross-sectional analysis is based on data from the baseline visit of the German national COPD cohort COSYCONET (*German****CO****PD and****Sy****stemic Consequences -****Co****morbidities****Net****work*). In brief, COSYCONET recruited 2,741 patients ≥ 40 years with physician-diagnosed COPD by outpatient and inpatient healthcare providers, patient groups, and media campaigns and examined them in 31 study centers all over Germany between September 2010 and December 2013. Exclusion criteria were previous lung transplantation or lung volume reduction surgery and lung malignancies. All participants were clinically stable defined as no moderate or severe exacerbations for at least 4 weeks at the time of enrolment. Details on the cohort have been published elsewhere [9, 10].

### Lung function test and definition of COPD

Standardized spirometry was performed in the COSYCONET cohort after bronchodilation with 400 μg salbutamol and 80 μg ipratropium bromide. COPD was defined as FEV_1_/FVC < 0.7 according to the GOLD criteria [1]. Patients were classified as grade 1 with FEV_1_ % pred. ≥ 80, grade 2 with 50 ≤ FEV_1_ % pred. < 80, grade 3 with 30 ≤ FEV_1_ % pred. < 50 and grade 4 with FEV_1_ % pred. < 30, with predicted values based on reference equations from the Global Lung Initiative (GLI) [11]. 430 participants from the cohort reporting a physician-diagnosed COPD but with FEV_1_/FVC ≥ 0.7 were excluded from this analysis as well as 20 participants with incomplete or missing lung function data.

The BODE index [12] as established multidimensional grading system in COPD was calculated in addition based on body mass index (BMI, defined as weight in kilograms/height in squared meters), FEV_1_ % pred., the 5-point modified Medical Research Council (mMRC) dyspnea scale and the six-minute walking test which was performed according to the criteria of the American Thoracic Society [13]. The BODE index ranges from 0 to 10 points with higher values indicating worse health.

### HRQL assessment

HRQL was assessed by self-administered questionnaires: The St. George’s Respiratory Questionnaire in its COPD-specific version (SGRQ-C) [14] and the COPD Assessment Test (CAT) were used as disease-specific HRQL instruments, and the EuroQol 5 dimensions (EQ-5D-3 L) as a generic HRQL instrument.

The SGRQ-C with 40 items provides three component scores for symptoms, activity and impact, and a total score. Each score ranges from 0 (no impairment) to 100 (worst possible). A difference of 4 unit points is considered the minimum clinically important difference (MCID) [15].

The CAT is an 8-item, short and validated tool for the assessment and monitoring of COPD [16]. Symptoms are assessed on a scale from 0 to 5. The total score ranges from 0 to 40 with higher scores representing worse health status. A MCID of 2 points has been proposed [17].

The EQ-5D-3 L questionnaire is a preference-based HRQL instrument with two parts. Part 1, the descriptive section, covers five dimensions of health: mobility, self-care, usual activities, pain/discomfort, and anxiety/depression with three levels per item (no problems, some problems, and extreme problems). An index-based utility score (EQ-5D utility) ranging from 0 to 1 can be calculated via a scoring algorithm which is based on valuations of representative general population samples. We used the German time-trade-off tariff by Greiner et al. for scoring [18]. A MCID for the utility score in COPD patients has not yet been established. Based on other diseases, it may range between 0.08-0.10 [19, 20]. Part 2, the valuation section, comprises a Visual Analog Scale (VAS) for valuing health states on a rating scale from 0 (worst imaginable health state) to 100 (best imaginable health state). A MCID of 8 points has been suggested in COPD patients with moderate to severe disease [21].

All three HRQL instruments are designed to assess current health status without specifying a recall period.

### Comorbidities and covariables

Self-reported information on physician-diagnosed comorbid conditions was systematically obtained in semi-structured interviews. We assessed 33 of these comorbid conditions. The number of comorbid conditions was summarized as a simple count. This approach has been shown to be a good proxy for the burden of comorbidities [22]. For defining groups with a low or a high number of comorbidities, the median number of comorbidities (≤3 vs. >3) was used as cut-off. A list of all comorbidities considered can be found in the (Additional file 1: Figure S1).

Information on age, sex, school education (basic (≤9 years), secondary (10–11 years), higher (≥12 years)), and smoking status (current, former, and never smoker) was collected by standardized questionnaires. BMI was measured at the study centers and classified as normal weight (18.5 ≤ BMI < 25), overweight (25 ≤ BMI < 30), obese (BMI ≥ 30), and underweight (BMI < 18.5).

### Statistical analysis

Characteristics of COPD patients in different grades were compared using analysis of variance (ANOVA) for continuous variables and Chi^2^-tests for categorical variables. For each HRQL instrument, the proportion of participants with the worst or best possible health state was determined to assess the presence of floor or ceiling effects. Bivariate correlations between the HRQL instruments and with clinical COPD measures were quantified by Spearman’s rank coefficient.

To assess the association of COPD grades 1–4 with HRQL and the discriminative ability of HRQL instruments, linear regression models were performed adjusting for age, sex, education, smoking status, BMI, and low vs. high number of comorbidities. The number of comorbid conditions as a continuous variable was considered in a sensitivity analysis instead of the dichotomized variable. Adjusted mean HRQL scores resulting from the regression models were reported for COPD grade 1–4 as well as for the group with a low or high number of comorbidities. Standardized mean differences were calculated as the mean adjusted difference between two COPD grades divided by their pooled (unadjusted) standard deviation (SD) [23] in order to assess the magnitude of the difference and to judge the ability of HRQL instruments for discrimination. In additional models, non-additive effects of COPD and comorbidity were checked by interaction terms between COPD grades and low or high number of comorbidities. Pseudo-R^2^ according to Cox & Snell [24] were calculated to compare the additional variance explained by COPD grade and/or low vs. high number of comorbidities in models for all HRQL instruments.

Further models were calculated controlling for all single comorbidities instead of a comorbidity count to identify the most important comorbid conditions for each HRQL instrument, again adjusted for age, sex, education, smoking status, and BMI.

Statistical analyses were performed using SAS software (SAS Institute Inc., Cary, NC, USA, version 9.3), and *p*-values of 0.05 or less were considered to be statistically significant.

## Results

The characteristics of the 2,291 COPD patients in grade 1 to 4 included for analysis are shown in Table 1. The majority of patients was male with a mean age of 65 years, FEV_1_ of 52.5 % predicted, BODE index of 2.5, and 3.7 self-reported comorbidities. Most of the COPD patients were in GOLD grade 2 (42 %) and 3 (38 %). COPD grades were comparable in their gender distribution while they differed significantly regarding age, education, smoking status, BMI, and the number of comorbidities.

**Table 1:** Characteristics of the study population and unadjusted means of HRQL

		Total sample	COPD grade 1	COPD grade 2	COPD grade 3	COPD grade 4	*p*-value
n (%)		2,291	206 (9.0)	962 (42.0)	874 (38.1)	249 (10.9)	
Age	Mean age (SD)	65.1 (8.4)	66.2 (8.7)	65.7 (8.5)	65.0 (8.2)	62.1 (8.0)	<0.0001
	% < 55 years (n)	11.4 (262)	9.7 (20)	10.3 (99)	11.2 (98)	18.1 (45)	<0.0001
	% 55 – 64 years (n)	33.6 (769)	27.2 (56)	31.7 (305)	34.3 (300)	43.4 (108)	
	% 65 – 74 years (n)	43.2 (989)	48.1 (99)	44.5 (428)	43.8 (383)	31.7 (79)	
	% > 74 years (n)	11.8 (271)	15.1 (31)	13.5 (130)	10.6 (93)	6.8 (17)	
Sex	% male (n)	60.9 (1,396)	60.2 (124)	60.2 (579)	61.0 (533)	64.3 (160)	0.70
School education	% basic education (n)	55.4 (1,270)	48.5 (100)	52.2 (502)	60.0 (524)	57.8 (144)	0.0002
	% secondary education (n)	27.1 (620)	25.2 (52)	28.6 (275)	25.2 (220)	29.3 (73)	
	% higher education (n)	17.5 (401)	26.2 (54)	19.2 (185)	14.9 (130)	12.9 (32)	
Smoking status	% smokers (n)	24.7 (565)	30.1 (62)	28.8 (277)	21.7 (190)	14.5 (36)	<0.0001
	% former smokers (n)	68.8 (1,576)	62.6 (129)	63.6 (612)	72.7 (635)	80.3 (200)	
	% never smokers (n)	6.6 (150)	7.3 (15)	7.6 (73)	5.6 (49)	5.2 (13)	
BMI	Mean BMI (SD) ^a^	26.7 (5.2)	26.6 (4.6)	27.4 (5.1)	26.4 (5.4)	24.4 (5.0)	<0.0001
	% normal weight (n)	37.1 (848)	36.4 (75)	32.6 (313)	38.5 (336)	49.8(124)	<0.0001
	% overweight (n)	37.1 (848)	42.2 (87)	38.9 (374)	36.2 (316)	28.5 (71)	
	% obese (n)	22.4 (512)	19.9 (41)	27.0 (259)	20.9 (182)	12.1 (30)	
	% underweight (n)	3.5 (81)	1.5 (3)	1.6 (15)	4.5 (39)	9.6 (24)	
Mean FEV_1_ % pred.		52.5 (18.6)	88.6 (8.1)	62.7 (8.3)	40.7 (5.6)	24.8 (3.9)	<0.0001
Mean BODE index (SD) ^b^		2.5 (2.0)	0.4 (0.7)	1.3 (1.2)	3.6 (1.5)	5.3 (1.6)	<0.0001
Comorbidities	Mean number (SD)	3.7 (2.6)	4.1 (2.7)	3.9 (2.6)	3.7 (2.6)	3.1 (2.3)	<0.0001
	% low number of comorbidities (n)	52.6 (1,206)	48.5 (100)	50.6 (487)	52.9 (462)	63.1 (157)	0.003
	% high number of comorbidities (n)	47.4 (1,085)	51.5 (106)	49.4 (475)	47.1 (412)	37.0 (92)	
HRQL	EQ-5D utility (SD) ^c^	0.82 (0.20)	0.85 (0.18)	0.84 (0.19)	0.81 (0.21)	0.74 (0.24)	<0.0001
	EQ-5D VAS (SD) ^d^	56.5 (19.6)	66.9 (17.4)	61.0 (18.8)	52.2 (18.8)	45.5 (17.8)	<0.0001
	CAT score (SD) ^e^	18.2 (7.4)	14.2 (6.8)	16.9 (7.1)	19.4 (7.2)	22.1 (6.8)	<0.0001
	SGRQ score (SD) ^f^	43.6 (20.0)	28.0 (15.8)	38.7 (19.1)	48.6 (17.9)	58.4 (18.0)	<0.0001

^a^ *n* = 2,289, ^b^ *n* = 2,197, ^c^ *n* = 2,277, ^d^ *n* = 2,266, ^e^ *n* = 2,276, ^f^ *n* = 2,272

### Completeness of HRQL questionnaires, floor- and ceiling effects and correlations

Completeness of questions was >98.8 % in all HRQL instruments. COPD-specific HRQL instruments and EQ-5D VAS showed no floor or ceiling effects. Regarding the EQ-5D descriptive section, 18 % of patients reported the best health state (11111).

With regard to the relationship between the HRQL instruments, correlations between EQ-5D utility index and disease-specific HRQL scores were moderate (rho = -0.56 for both CAT and SGRQ). Compared to the EQ-5D utility score, the VAS showed a stronger correlation with the CAT score (rho = -0.62) and the SGRQ total score (rho = -0.65). Regarding the correlations of HRQL instruments with measures of disease severity, the highest correlation was found between the SGRQ total or activity score and the BODE index (rho = 0.61 or rho = 0.67). COPD grade correlated best with the SGRQ total score (rho = 0.40) and activity score (rho = 0.46). The BODE index correlated better with HRQL instruments than GOLD grade. For the number of comorbid conditions, correlation was best for EQ-5D utility (rho = -0.29).

An overview of correlations can be found in Additional file 1: Table S1.

### HRQL scores in different COPD grades

In an unadjusted comparison, higher COPD grades showed gradually worse generic and disease-specific HRQL scores (Table 1). Individual variability within the COPD grades was high (Additional file 1: Figure S2).

Table 2 shows the association of COPD grades with HRQL measures after adjusting for possible confounders. Patients in COPD grades 2–4 had significantly worse HRQL across all instruments and scales compared to COPD grade 1, except for the EQ-5D utility. For example, COPD grade 2 was associated with a 5.7 point reduction of the ED-5D VAS (*p* < 0.0001). This reduction increased to 22.1 points for COPD grade 4 (*p* < 0.0001).

**Table 2:** Results of regression analyses

		EQ-5D utility		EQ-5D VAS		CAT score		SGRQ total score	
		Estimate	*p*-value	Estimate	*p*-value	Estimate	*p*-value	Estimate	*p*-value
COPD	Grade 4	-0.13	<0.0001	-22.11	<0.0001	7.92	<0.0001	31.71	<0.0001
	Grade 3	-0.04	0.005	-14.61	<0.0001	4.98	<0.0001	20.41	<0.0001
	Grade 2	-0.006	0.69	-5.67	<0.0001	2.47	<0.0001	10.28	<0.0001
	Grade 1	ref.		ref.		ref.		ref.	
Comorbidities	High number	-0.09	<0.0001	-5.75	<0.0001	2.91	<0.0001	7.90	<0.0001
	Low number	ref.		ref.		ref.		ref.	
Age	>74 years	-0.01	0.59	-1.44	0.38	-2.05	0.001	1.20	0.45
	65 – 74 years	0.01	0.36	1.04	0.43	-1.55	0.002	0.23	0.86
	55 – 64 years	-0.001	0.97	0.26	0.84	-0.60	0.23	0.26	0.84
	<55 years	ref.		ref.		ref.		ref.	
Sex	Female	-0.02	0.06	1.53	0.06	0.16	0.60	0.90	0.25
	Male	ref.		ref.		ref.		ref.	
School education	Higher	0.05	<0.0001	3.08	0.003	-1.77	<0.0001	-4.58	<0.0001
	Secondary	0.02	0.06	3.10	0.001	-0.88	0.01	-3.26	0.0002
	Basic	ref.		ref.		ref.		ref.	
Smoking status	Current smoker	-0.01	0.46	-0.13	0.94	0.74	0.25	1.19	0.47
	Former smoker	-0.02	0.29	0.29	0.85	0.18	0.77	1.45	0.34
	Never smoker	ref.		ref.		ref.		ref.	
BMI	Overweight	-0.03	0.007	-1.53	0.09	0.14	0.68	1.50	0.08
	Obese	-0.06	<0.0001	-4.68	<0.0001	1.64	<0.0001	6.40	<0.0001
	Underweight	0.002	0.93	-5.59	0.01	0.87	0.28	1.97	0.34
	Normal weight	ref.		ref.		ref.		ref.	

**Fig. 1:**
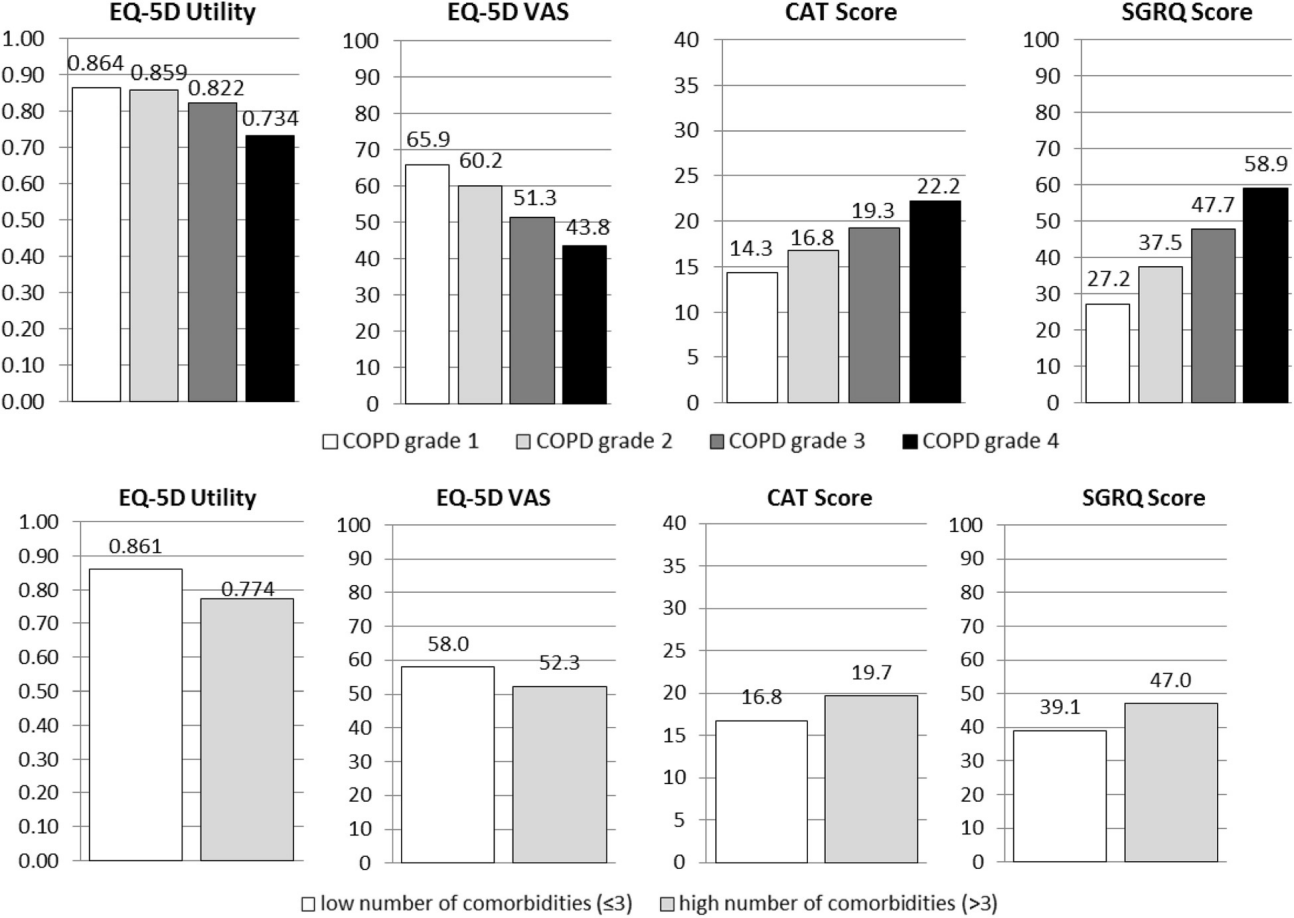
Adjusted mean EQ-5D utilities, EQ-5D VAS, CAT score, SGRQ score by COPD grade 1-4 and comorbidity group

Adjusted mean HRQL scores by COPD grade are illustrated in Fig. 1. There were clear graduations between COPD grades in all instruments except for the EQ-5D utility in grade 1 and 2. Especially, the SGRQ score seemed to increase linearly with disease grade. When considering the number of comorbid conditions as a continuous instead of a dichotomized variable, the adjusted means per COPD grade were virtually unchanged.

**Fig. 2:**
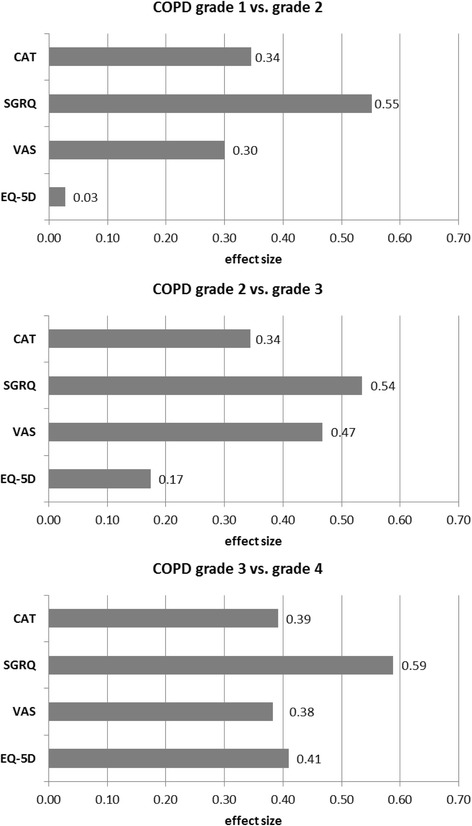
Standardized absolute mean differences between COPD grades

Figure 2 shows the calculated effect sizes to assess the importance of the differences between COPD grades. The SGRQ total score showed the best discrimination between all grades of COPD. The CAT score and the VAS could also equally differentiate between COPD grades both in early and advanced disease grades, whereas the EQ-5D utility differentiated much better between COPD patients in grade 3 and 4 than in earlier grades.

### HRQL instruments and comorbid conditions

Having 4 or more comorbidities was significantly associated with worse HRQL in all instruments, as shown in Table 2. As to EQ-5D VAS and CAT, the effect estimate of a high number of comorbidities compared to a low number of comorbidities was approximately of the same size than the difference between COPD grade 1 and 2. For the SGRQ, the comorbidity estimate was lower than the estimate of COPD grade 2, but still higher than the suggested MCID. Compared to the estimates of the COPD grades, the effect estimate of high comorbidity was highest as to the EQ-5D utility, ranking between grade 3 and 4. Adjusted mean HRQL scores by comorbidity group are illustrated in Fig. 1. Combining with disease severity, adjusted mean HRQL scores for comorbidity-group by COPD grade are reported in the Additional file 1: Table S2.

In additional models including interaction terms, the effects of COPD grades and comorbidity on HRQL measures were found to be additive, as all interaction terms were non-significant (estimates shown in Additional file 1: Table S3).

When controlling for COPD grades in addition to age, sex, education, smoking status and BMI in regression models, the increase of explained variance was higher for the VAS, CAT, and SGRQ score than for the EQ-5D utility. In contrast, controlling for a low vs. high number of comorbidities resulted in a higher percentage of explained variance for the EQ-5D utility than for VAS, CAT, and SGRQ. Details on the values of R^2^ are shown in Table 3.

**Table 3:** Comparison of pseudo-R-squared^a^ of different models (percentage of variance explained by different models)

Models considering…	EQ-5D utility	EQ-5D VAS	CAT score	SGRQ total score
Age, sex, education, smoking status, BMI category	0.034	0.029	0.041	0.043
Age, sex, education, smoking status, BMI category and COPD grade	0.063	0.125	0.112	0.201
Age, sex, education, smoking status, BMI category and low/high number of comorbidities	0.074	0.048	0.074	0.075
Age, sex, education, smoking status, BMI category, COPD grade and low/high number of comorbidities	0.106	0.145	0.149	0.238

^a^ R^2^ according to Cox & Snell [24]

When considering single comorbidities instead of a comorbidity count, psychological disorders comprising depression and anxiety showed one of the highest effect estimates in all models, both for generic and disease-specific instruments (Table 4). Furthermore, peripheral artery disease (PAD) ranked among the top 5 of comorbidities with negative effects on HRQL in all models as well as sleep apnea except for the EQ-5D utility. Obesity was also significantly associated with a considerably reduced HRQL in all instruments.

**Table 4:** Effect estimates of top 5 single comorbid conditions with significant negative association with HRQL scores

Rank	EQ-5D utility			EQ-5D VAS			CAT			SGRQ		
1	Psychiatric disorder ^a^	-0.09	(*p* < 0.0001)	Brain deficiency ^b^	-5.9	(*p* = 0.001)	Psychiatric disorder ^a^	2.5	(*p* < 0.0001)	Psychiatric disorder ^a^	7.2	(*p* < 0.0001)
2	Arthritis	-0.06	(*p* < 0.0001)	Psychiatric disorder ^a^	-5.3	(*p* < 0.0001)	Asthma	1.7	(*p* < 0.0001)	PAD	5.0	(*p* < 0.0001)
3	PAD	-0.05	(*p* < 0.0001)	PAD	-3.4	(*p* = 0.005)	Sleep apnea	1.5	(*p* = 0.002)	Brain deficiency	4.6	(*p* = 0.005)
4	Arthrosis	-0.05	(*p* = 0.0003)	Asthma	-3.4	(*p* = 0.001)	Heart disease ^c^	1.5	(*p* < 0.0001)	Sleep apnea	4.3	(*p* = 0.0004)
5	Migraine	-0.05	(*p* < 0.0001)	Sleep apnea	-2.8	(p = 0.03)	PAD	1.4	(*p* = 0.002)	Heart disease ^c^	4.1	(*p* < 0.0001)

all models adjusted for age, sex, school education, smoking status, BMI category, and GOLD grade

^a^comprising anxiety, depression, psychoses

^b^comprising weakness of memory, disorientation, confusion

^c^comprising cardiac arrhythmia, cardiac insufficiency, narrow coronary vessel, angina pectoris

For the disease-specific instruments as well as the VAS, the effect estimates of all single comorbid conditions were comparable to or smaller than the estimate of COPD grade 2 (Table 4). For the EQ-5D utility, all estimates of the 5 comorbidities with the largest effects ranged between the effect of COPD grade 3 (-0.05) and grade 4 (-0.14).

## Discussion

This study performed a comparison of the most frequently used generic and disease-specific HRQL instruments in a large cohort of COPD patients in all levels of airflow limitation with particular attention on the association with a comprehensive list of comorbid conditions.

Our study confirms that mean scores of SGRQ, CAT, EQ-5D utility, and EQ-5D VAS worsen with increasing COPD grade, despite high variability within each grade. EQ-5D utility and VAS as generic HRQL instruments and SGRQ and CAT as disease-specific instruments were found able to discriminate between COPD grades, with some limitations for the EQ-5D utility in mild COPD. Utility results which are based on population preferences and therefore introduce external HRQL valuation put the highest weight on comorbid conditions. However, disease-specific HRQL instruments also reflected the burden of comorbid conditions. Non-additive effects of COPD and comorbidity were not observed. When focusing on single comorbid conditions, psychiatric disorders and peripheral artery disease ranked among the top 5 comorbid conditions with negative associations with HRQL for all instruments. Sleep apnoea, brain deficiency, asthma, and heart disease were also important contributors to low HRQL.

Regarding mean HRQL scores per COPD grade or correlation of disease-specific and generic HRQL measures, our results are in line with previous studies. Despite the differences in underlying populations, the mean CAT scores of our study fit very well in the range of means reported in a systematic review on studies using the CAT by Gupta et al. [25]. In a small sample of US veterans in COPD grades 1–4 (*n* = 120), Pickard et al. reported correlations between EQ-5D utility or VAS and SGRQ total score similar to our findings [26]. The EQ-5D VAS means, which were considerably higher than in our study, differed between COPD grades 1–4, while EQ-5D utilities did not. In line with our results, best discriminative properties between COPD grades were reported for the SGRQ in this study.

Our results showed clear HRQL differences between COPD grade 1 and 2 (except for EQ-5D utility), while some previous studies did not observe HRQL differences between milder COPD grades. E.g., Jones et al. did not find differences in SGRQ total score and the generic SF-12 questionnaire between primary care COPD patients in grade 1 and 2. This might be due to the fact that reported SGRQ means for grade 1 were more than 10 points higher than in our sample, while mean values for grades 2, 3 and 4 as reported in Jones et al. were comparable to our results [27]. In the same population, Jones et al. did not find significant differences in CAT scores between COPD grade 1 and 2 [28]. A possible explanation for this lack of differences between these grade 1 and 2 patients may be that symptoms and HRQL impairment are important drivers for the patients to see their primary care physician.

For health-economic evaluations in COPD, it is important to know the discriminative abilities of the EQ-5D questionnaire because it is frequently applied for determining utilities needed for the calculations of quality-adjusted life years (QALYs) [29]. Based on data from UPLIFT, a large COPD trial covering 13 countries, Rutten-van Mölken et al. investigated discriminative abilities of the EQ-5D questionnaire in COPD patients [29]. This study found better discriminative properties of the EQ-5D utility in higher COPD grades, but did not include COPD grade 1. When comparing EQ-5D utilities, it has to be kept in mind that utilities depend on the underlying country-specific tariff and methods (time trade off vs. standard gamble) used for their calculation. Furthermore, ceiling effects may limit the sensitivity of the utility.

There seems to be a consensus that COPD patients with a higher number of comorbid conditions have a worse HRQL [27, 28, 30–34]. Several previous studies supplied evidence that comorbidities in patients with COPD are associated with worse COPD-specific HRQL scores [6, 27, 28, 31, 35, 36]. In a hospital-based COPD cohort, Koskela et al. also found that a generic HRQL instrument better captured the effects of comorbid conditions but that these conditions were also associated with COPD-specific HRQL [3]. For COPD patients with three or more comorbid conditions, Jones et al. reported significantly worse CAT (+2.5 units) [28] and SGRQ scores (approx. +8 units) [27] than for patients without or with 1–2 comorbidities. Despite referring to different comorbid conditions, these extra units correspond well to the estimates of high comorbidity found in our study.

When focusing on single comorbid conditions, especially mental health problems are often reported contributors to decreased HRQL in COPD [3, 5, 6, 31, 34, 37]. This was confirmed by our analysis. In general, a direct comparison of the effects of single comorbidities on HRQL in COPD patients is often hampered by different conditions considered and diverging definitions. Depression, anxiety, and PAD which we identified as the most important comorbidities in our study, were also included in the recently published COMCOLD index that identified five comorbidities with the highest effect on the VAS [38]. Further comparison with the two additional comorbidities of this index (symptomatic heart disease and cerebrovascular disease) was not possible due to differences in definitions.

Urff et al. reported that COPD patients with depression or heart failure had considerably lower disease-specific HRQL as measured by the Clinical COPD Questionnaire (CCQ) [36] and explained this finding by overlapping symptoms of COPD and depression or heart failure. Having the items of EQ-5D, CAT, and SGRQ in mind, another reason could be that there is an overlap of these instruments with regard to psychological and mobility aspects of health. Therefore, it is not surprising that especially psychological disorders and PAD showed the strongest associations with the HRQL scores in our analysis. We did not consider dyspnea and exacerbation frequency in our multivariate analysis. As the CAT and SGRQ include dyspnea and symptoms of exacerbations already in their items, controlling for these factors as explanatory variables would cause problems of circularity. The finding that the multidimensional BODE index showed higher correlation with HRQL scores than COPD grades is in line with this reasoning because BODE includes mMRC as a measure of dyspnea and the 6-minute-walking-test as a measure of physical capacity.

Further explanations for the ability of disease-specific HRQL instruments to reflect the burden of comorbidity need to be addressed in future research.

Several strengths of our study have to be considered. Based on a large patient sample comprising all COPD grades, our analysis had sufficient power to detect differences between grades. A comprehensive list of comorbid conditions was available and possible interactions between COPD and comorbidity were analyzed. Therefore, the results of our observational study might be more generalizable than results from clinical trials where patients with comorbidities are often excluded.

There are, however, also potential limitations: First, since only cross-sectional data were available, we were not able to investigate the responsiveness of HRQL instruments or sensitivity to change over time. Second, data on physician-diagnosed comorbidities were self-reported which might limit their validity, and no information on the severity of comorbid conditions was available. Furthermore, comorbid conditions were summarized and dichotomized without weighting. However, this approach has been shown to perform just as well as weighted scores to quantify the impact of comorbidities on disease-specific HRQL in COPD patients [22].

Nevertheless, from a research perspective, our direct comparison of HRQL instruments did not show a clear ranking. The decision for a generic or disease-specific instrument must be based on the purpose of HRQL measurement. Cross-sectional comparisons of COPD patients (with or without control subjects) or a longitudinal monitoring of disease progression pose different demands on HRQL instruments. Researchers are responsible to ensure that the chosen HRQL instrument addresses the problems of the research questions. While generic instruments allow comparisons of groups with different conditions, may support the identification of unexpected HRQL issues, and are need for the calculation of QALYs in economic evaluations, disease-specific instruments are more likely to detect small, but relevant changes [39]. Furthermore, by focusing on relevant dimensions of the disease, disease-specific instruments may have a higher acceptability among patients and thus increased responsiveness.

However, we have shown that the effect of comorbid conditions on HRQL was both apparent in generic and to a smaller degree in COPD-specific HRQL measures. This finding is consistent with studies from other diseases [40]. To cover all aspects of HRQL in COPD, both generic and disease-specific instruments should be used in the ideal case. With regard to economic evaluations, further research is needed towards the discriminative properties of the new EQ-5D 5-level version in COPD.

From a clinical perspective, our results underline the need for a holistic approach for COPD healthcare with a mandatory screening for comorbidities, assessment of HRQL and in consequence a multidisciplinary treatment as generic and also COPD-specific health status could be improved [41].

## Conclusion

This large observational study confirms that HRQL is considerably impaired in COPD patients and worsens with disease deterioration and higher comorbidity. Findings underline the need of diagnosing and treating comorbid conditions in COPD patients as generic and also COPD-specific health status could be improved. Results may further be useful for choosing HRQL instruments in future clinical studies, and for parameterizing decision-analytic COPD models.

## Ethics approval and consent to participate

The COSYCONET study complies with the Declaration of Helsinki and Good Clinical Practice Guidelines and has been approved by the ethics committee of the medical faculty of the Philipps-Universität Marburg, the local ethics committees of the participating centers (a list of all participating study centers can be found here: http://www.asconet.net/html/cosyconet/studzent) and by the concerned data security authority (data security agency of the federal states of Hesse, Baden-Württemberg, Lower-Saxony, and Saarland). All participants provided written informed consent.

## Availability of data and materials

The full dataset supporting the conclusions of this article is available upon request and application from the Competence Network Asthma and COPD (ASCONET, http://www.asconet.net/html/cosyconet/projects).

## Consent for publication

Not applicable.

## Additional file

Additional file 1: Table S1. Correlations between HRQL instruments and with GOLD grade and BODE as clinical measures: Spearman Correlation Coefficients. **Table S2.** Adjusted mean EQ-5D utilities, EQ-5D VAS, CAT score, SGRQ total scores for COPD grade 1–4 stratified for group with low (≤3) or high (>3) number of comorbidities. **Table S3.** Results of regression models considering interactions between COPD grades and low/high number of comorbidity. **Figure S1.** Lifetime prevalence of self-reported comorbidities (%) in the study population. **Figure S2.** HRQL scores by FEV1 % pred.: non-parametric quantile regression: quantile fit plots for FEV_1_ % pred. (DOC 258 kb)

## Abbreviations

ANOVA: analysis of variance
BMI: body mass index
BODE: body-mass index, airflow obstruction, dyspnea and exercise capacity index
CAT: COPD assessment test
COPD: chronic obstructive pulmonary disease
COSYCONET: German COPD and Systemic Consequences - Comorbidities Network
EQ-5D-3 L: EuroQol 5 dimensions questionnaire with 3 levels
FEV_1_: forced expiratory volume in 1 second
FVC: forced vital capacity
HRQL: health-related quality of life
MCID: minimum clinically important difference
PAD: peripheral artery disease
QALY: quality-adjusted life year
SD: standard deviation
SGRQ-C: St. George’s Respiratory Questionnaire, COPD-specific version
VAS: visual analog scale
