# Supplementary material for: Assessing health-related quality of life in COPD: comparing generic and disease-specific instruments with focus on comorbidities
Source: BMC Pulm Med. 2016 May 10;16:70. doi: 10.1186/s12890-016-0238-9 (PMC4862227; doi:10.1186/s12890-016-0238-9)

**Additional file 1:**

**Table S1: Correlations between HRQL instruments and with GOLD grade and BODE as clinical measures: Spearman Correlation Coefficients**

| **Spearman Correlation Coefficients** | | | | | | | | | | |
| --- | --- | --- | --- | --- | --- | --- | --- | --- | --- | --- |
| **Prob > |r| under H0: Rho=0** | | | | | | | | | | |
|  | **EQ-5D utility** | **EQ-5D VAS** | **CAT Score** | **SGRQ Total Score** | **SGRQ Symptoms Score** | **SGRQ Activity Score** | **SGRQ Impact Score** | **BODE index** | **COPD grade** | **Number of comor-bidities** |
| **EQ-5D utility** | 1 | 0.47 | -0.56 | -0.56 | -0.38 | -0.50 | -0.53 | -0.33 | -0.15 | -0.29 |
| **EQ-5D VAS** | 0.47 | 1 | -0.62 | -0.65 | -0.47 | -0.59 | -0.61 | -0.48 | -0.32 | -0.16 |
| **CAT Score** | -0.56 | -0.62 | 1 | 0.78 | 0.67 | 0.64 | 0.74 | 0.46 | 0.27 | 0.21 |
| **SGRQ total score** | -0.56 | -0.65 | 0.78 | 1 | 0.73 | 0.88 | 0.95 | 0.61 | 0.40 | 0.21 |
| **SGRQ Symptoms Score** | -0.38 | -0.47 | 0.67 | 0.73 | 1 | 0.51 | 0.65 | 0.36 | 0.23 | 0.18 |
| **SGRQ Activity Score** | -0.50 | -0.59 | 0.64 | 0.88 | 0.51 | 1 | 0.74 | 0.67 | 0.46 | 0.18 |
| **SGRQ Impact Score** | -0.53 | -0.61 | 0.74 | 0.95 | 0.65 | 0.74 | 1 | 0.52 | 0.32 | 0.21 |
| **BODE index** | -0.33 | -0.48 | 0.46 | 0.61 | 0.36 | 0.67 | 0.52 | 1 | 0.79 | 0.05 ^a^ |
| **COPD grade** | -0.15 | -0.32 | 0.27 | 0.40 | 0.23 | 0.46 | 0.32 | 0.79 | 1 | -0.09 |
| **Number of comor-bidities** | -0.29 | -0.16 | 0.21 | 0.21 | 0.18 | 0.18 | 0.21 | 0.05 ^a^ | -0.09 | 1 |

all p<0.0001, except ^a^ p=0.13

**Table S2: Adjusted mean EQ-5D utilities, EQ-5D VAS, CAT score, SGRQ total scores for COPD grade 1-4 stratified for group with low (≤3) or high (>3) number of comorbidities**

|  | **EQ-5D utility** | | **EQ-5D VAS** | | **CAT Score** | | **SGRQ Total Score** | |
| --- | --- | --- | --- | --- | --- | --- | --- | --- |
|  | *low number of comorbidities* | *high number of comorbidities* | *low number of comorbidities* | *high number of comorbidities* | *low number of comorbidities* | *high number of comorbidities* | *low number of comorbidities* | *high number of comorbidities* |
| COPD grade 1 | 0.900 | 0.825 | 69.4 | 61.9 | 12.3 | 16.6 | 23.5 | 32.1 |
| COPD grade 2 | 0.895 | 0.817 | 63.4 | 56.3 | 15.3 | 18.6 | 33.4 | 42.8 |
| COPD grade 3 | 0.855 | 0.785 | 53.9 | 48.1 | 18.0 | 21.0 | 44.4 | 51.9 |
| COPD grade 4 | 0.770 | 0.693 | 46.5 | 40.4 | 21.3 | 23.4 | 56.4 | 62.3 |

all models adjusted for age, sex, school education, smoking status, BMI category

**Table S3: Results of regression models considering interactions between COPD grades and low/high number of comorbidity**

|  |  | |  | **EQ-5D utility** | | | **EQ-5D VAS** | | **CAT Score** | | **SGRQ  Total Score** | |
| --- | --- | --- | --- | --- | --- | --- | --- | --- | --- | --- | --- | --- |
|  | | |  | **estimate** | | ***p-value*** | **estimate** | ***p-value*** | **estimate** | ***p-value*** | **estimate** | ***p-value*** |
| **COPD** | | **grade 4** | | | -0.13 | *<0.0001* | -22.86 | *<0.0001* | 8.86 | *<0.0001* | 32.67 | *<0.0001* |
|  | | **grade 3** | | | -0.04 | *0.05* | -15.45 | *<0.0001* | 5.60 | *<0.0001* | 20.89 | *<0.0001* |
|  | | **grade 2** | | | -0.002 | *0.91* | -5.79 | *0.004* | 2.96 | *<0.0001* | 9.79 | *<0.0001* |
|  | | **grade 1** | | | ref. |  | ref. |  | ref. |  | ref. |  |
| **comorbidities** | | **high number** | | | -0.08 | *0.002* | -6.64 | *0.009* | 3.98 | *<0.0001* | 8.10 | *0.0009* |
|  | | **low number** | | | ref. |  | ref. |  | ref. |  | ref. |  |
| **COPD grade 4 * high comorbidity** | | | | | -0.003 | *0.93* | 1.62 | *0.64* | -2.09 | *0.11* | -2.47 | *0.46* |
| **COPD grade 3 * high comorbidity** | | | | | 0.001 | *0.96* | 1.68 | *0.55* | -1.21 | *0.26* | -0.98 | *0.72* |
| **COPD grade 2 * high comorbidity** | | | | | -0.007 | *0.81* | 0.20 | *0.94* | -0.96 | *0.36* | 1.02 | *0.71* |
| **COPD grade 1 * high comorbidity** | | | | | ref. |  | ref. |  | ref. |  | ref. |  |

all models adjusted for age, sex, school education, smoking status, BMI category

**Figure S1: Lifetime prevalence of self-reported comorbidities (%) in the study population**


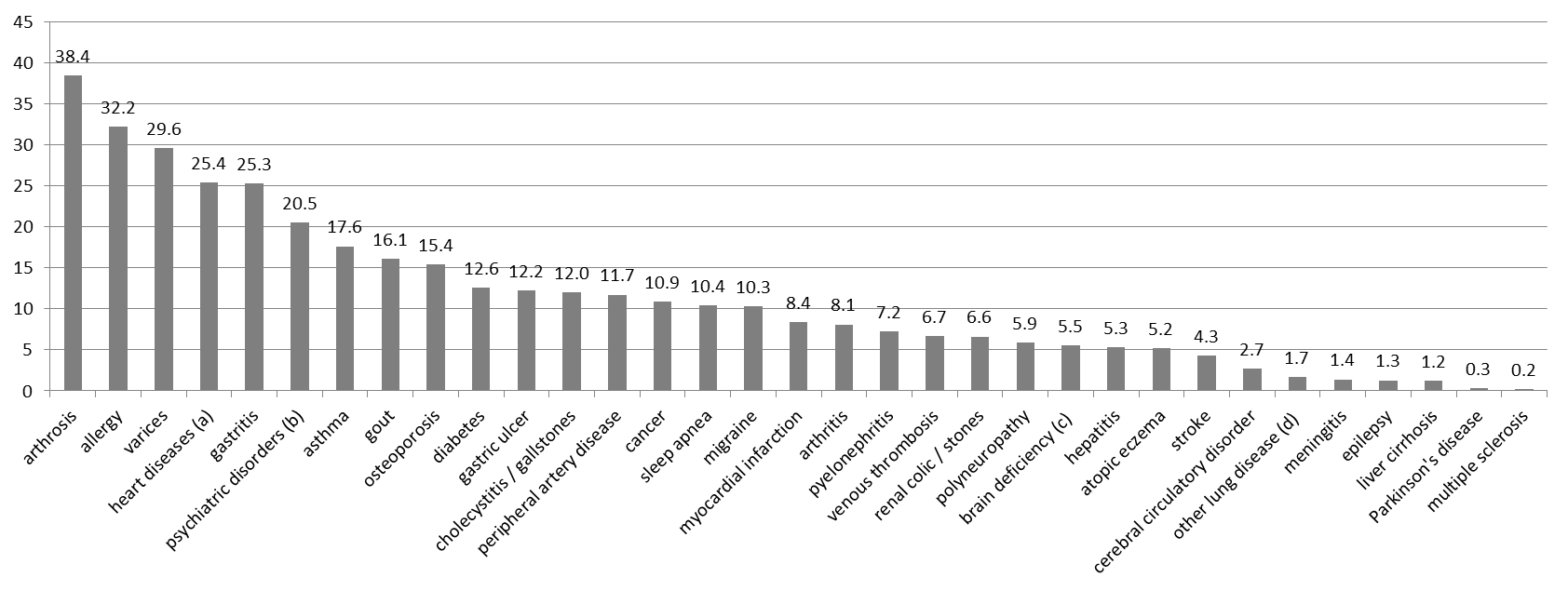


all confidence intervals have a width of less than 2.0.

^(a)^ comprising cardiac arrhythmia, cardiac insufficiency, narrow coronary vessel, angina pectoris

^(b)^ comprising anxiety, depression, psychoses

^(c)^ comprising weakness of memory, disorientation, confusion

^(d)^ comprising bronchiectasis, fibrosis, sarcoidosis

**Figure S2: HRQL scores by FEV1 % pred.: non-parametric quantile regression: quantile fit plots for FEV_1_ % pred.**


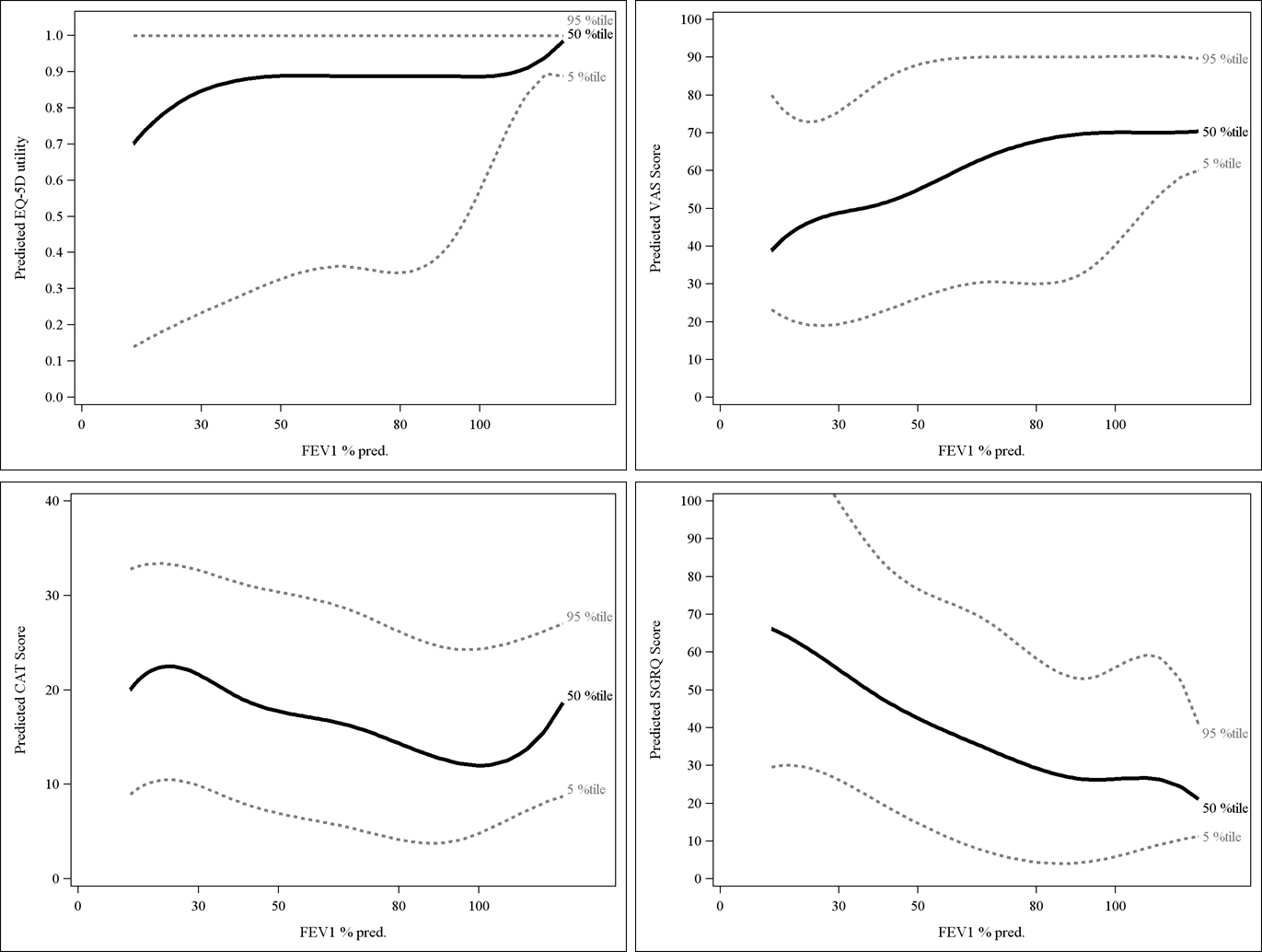

Supplement: Additional file 1: Table S1. — Correlations between HRQL instruments and with GOLD grade and BODE as clinical measures: Spearman Correlation Coefficients. Table S2. Adjusted mean EQ-5D utilities, EQ-5D VAS, CAT score, SGRQ total scores for COPD grade 1–4 stratified for group with low (≤3) or high (>3) number of comorbidities. Table S3. Results of regression models considering interactions between COPD grades and low/high number of comorbidity. Figure S1. Lifetime prevalence of self-reported comorbidities (%) in the study population. Figure S2. HRQL scores by FEV1 % pred.: non-parametric quantile regression: quantile fit plots for FEV1 % pred. (DOC 258 kb) [file 12890_2016_238_MOESM1_ESM.doc]
